# Deoxynivalenol induces apoptosis and autophagy in human prostate epithelial cells via PI3K/Akt signaling pathway

**DOI:** 10.1007/s00204-021-03176-z

**Published:** 2021-10-22

**Authors:** Karolina Kowalska, Marta Justyna Kozieł, Dominika Ewa Habrowska-Górczyńska, Kinga Anna Urbanek, Kamila Domińska, Agnieszka Wanda Piastowska-Ciesielska

**Affiliations:** 1 Medical University of Lodz, Department of Cell Cultures and Genomic Analysis, Zeligowskiego 7/9, 90-752 Lodz, Poland; 2 Medical University of Lodz, Department of Comparative Endocrinology, Zeligowskiego 7/9, 90-752 Lodz, Poland

**Keywords:** Deoxynivalenol, Prostate, Apoptosis, Autophagy, Oxidative stress

## Abstract

Phosphoinositide 3-kinase (PI3K)/protein kinase B (Akt) signaling pathway is one of the most deregulated signaling pathway in prostate cancer. It controls basic processes in cells: cell proliferation and death. Any disturbances in the balance between cell death and survival might result in carcinogenesis. Deoxynivalenol (DON) is one of the most common mycotoxins, a toxic metabolites of fungi, present in our everyday diet and feed. Although previous studies reported DON to induce oxidative stress, modulate steroidogenesis, DNA damage and cell cycle modulation triggering together its toxicity, its effect on normal prostate epithelial cells is not known. The aim of the study was to evaluate the effect of DON on the apoptosis and autophagy in normal prostate epithelial cells via modulation of PI3K/Akt signaling pathway. The results showed that DON in a dose of 30 µM and 10 µM induces oxidative stress, DNA damage and cell cycle arrest in G2/M cell cycle phase. The higher concentration of DON induces apoptosis, whereas lower one autophagy in PNT1A cells, indicating that modulation of PI3K/Akt by DON results in the induction of autophagy triggering apoptosis in normal prostate epithelial cells.

**Supplementary Information:**

The online version contains supplementary material available at 10.1007/s00204-021-03176-z.

## Introduction

Contamination of mycotoxins in feed and food constitutes a global health care problem and environmental issue since years (Awuchi et al. 2021). Deoxynivalenol (DON), also known as vomitoxin is a toxic metabolite of fungi produced mainly by *F. graminearum*. It contaminates cereal products and possesses the highest contamination level in feed and feedstuff products (Fisher et al. 2020). Moreover, DON is believed to be one of the commonest and most harmful mycotoxin, especially when taking into consideration its metabolites and modified forms present in the food chain (Warth et al. 2016).

The toxic effect of DON is associated with inhibition of protein synthesis by binding to ribosomes and induction of ribotoxic stress in cells (Ueno 1977). DON is also reported to induce oxidative stress (Pomothy et al. 2021), DNA damage (Mu et al. 2020), cell cycle arrest in G2/M cell cycle phase (Yuan et al. 2018), cell signaling deregulation and apoptosis (Mu et al. 2020). Chronic low doses of DON cause anorexia, immunotoxicity, placental toxicity and retardation of growth (Rotter et al. 1996). Although the toxic effect of DON is reported in many different cell lines, mostly in the intestinal cells, both porcine (Yu et al. 2021) and human (Pomothy et al. 2021) due to the first line of exposure to DON. DON was reported to modulate steroidogenesis (Urbanek et al. 2018), decreases the number of ovarian granule cells, inhibits proliferation and hormone production (Cortinovis et al. 2014). The induction of oxidative stress and apoptosis caused by DON was also present in piglets Sertoli cells (Cao et al. 2021). However, the effect of DON exposure on human prostate epithelial cells has not been evaluated yet, even the fact that DON was reported to induce reactive oxygen species (ROS) production and apoptosis in prostate cancer cells (Habrowska-Górczyńska et al. 2019).

Phosphoinositide 3-kinase (PI3K)/protein kinase B (Akt) signaling pathway is one of the most common dysregulated pathways in prostate cancer, due to the fact that regulates the basic processes in cells: proliferation and apoptosis. Its activation is associated with increased proliferation and decreased apoptosis of cells (Sreenivasulu et al. 2021). As a consequence several cancers, including prostate cancer, presents deregulation of PI3K/Akt signaling during tumor progression (Hinz and Jücker 2021). Any modulators of PI3K/Akt signaling pathway in normal prostate epithelial cells might participate in the process of carcinogenesis of prostate tissue. Previous studies showed that DON might induce apoptosis and autophagy in cells by modulation of PI3K/Akt/ mechanistic target of rapamycin (mTOR) signalling pathway in porcine jejunum epithelial cell line (Zhao et al. 2020). The phosphorylation of Akt was also observed after DON treatment in mouse skin cells (Mishra et al. 2014).

Thus, the aim of the study was to evaluate the effect of DON on normal prostate epithelial cells, with the focus of the induction of apoptosis and autophagy as well as involvement of PI3K/Akt signaling pathway in the effect of DON.

## Materials and methods

### Cell culture and treatment

Normal prostate epithelial cells (PNT1A) were obtained from the European Collection of Authenticated Cell Cultures (EACC, Sigma Aldrich, St. Louis, MO, USA). Cells were cultured in RPMI 1640 medium supplemented with 10% heat-inactivated Fetal Bovine Serum (FBS), 1 mM Sodium Pyruvate, 10 mM HEPES Buffer and antibiotics (all supplements were purchased from Thermo Fisher Scientific Inc, Waltham, MA, USA /Life technologies). RPMI without phenol red, FBS and antibiotics was used as experimental medium.

Stock solution of DON (Toronto Research Chemicals) was prepared in 99.8% ethanol. LY294002 was diluted in DMSO (14 mM). Final concentrations of DON and LY294002 were achieved in experimental medium. Cells treated only with LY294002 were used as a positive control, while cells treated with experimental medium as a negative control.

### Cell viability

Cells (0.01 × 10^6^) were seeded on 96-well plates. Next day, they were treated with experimental medium containing DON in concentration range 0.001 to 100 µM for 24, 48 and 72 h. 2 h before the end of the incubation time AlamarBlue reagent (Thermo Fisher Scientific Inc. Waltham, MA, USA/Life technologies) was added. EL808IU BioTek microplate reader (BioTek Instruments, Inc.) was used to measure absorbance at 570 nm and 600 nm. The results were expressed as the percentage of non-treated cells (Cnt).

Based on the obtained results two doses of DON were chosen for the rest of experiments with LY294002 (30 µM, 30 µM + LY, 10 µM, 10 µM + LY). For doses responsible for apoptosis 30 µM, 30 µM + LY were chosen, while for autophagy 10 µM, 10 µM + LY. As a positive control we used experimental medium with PI3K inhibitor (LY294002) as control we used non-treated cells. AlamarBlue assay was also performed for this configuration as described above.

### Oxidative stress

The number of ROS positive cells was determined with Muse® Oxidative Stress Kit (Luminex® Austin, Texas, United States) on Muse Cell Analyzer (Merck Millipore, Burlington, MA, USA). For this purpose, cells (0.3 × 10^6^) were seeded on 6-well plates and cultured in standard conditions. Then, cells were treated with experimental medium (30 µM, 30 µM + LY, 10 µM, 10 µM + LY, Cnt + LY and Cnt) for 24 h. After incubation time, oxidative stress assay was performed following manufacturer’s instructions. The experiment was carried out in three independent replicates.

### Autophagy

Cells (0.05 × 10^6^) were seeded intro 48-well plates. The next day, cells were induced with the experimental medium containing 10 µM, 10 µM + LY, Cnt + LY, Cnt, respectively. Then, cells were harvested and autophagy was assessed using Muse Autophagy LC3-Antibody Based Kit according to manufacturer’s instructions (Luminex® Austin, Texas, United States). The results are presented as Mean Autophagy Intensity.

### Apoptosis

An induction of apoptosis was evaluated with Annexin V & Dead Cell (Luminex® Austin, Texas, United States). For this purpose, cells in the number of 0.3 × 10^6^ were seeded on 6-well plates and induced with 30 µM, 30 µM + LY, Cnt + LY, Cnt for 24 h. After incubation, the procedure was performed accordingly to manufacture’s recommendations. The experiment was carried out in triplicate.

### Cell cycle analysis

The distribution of cell cycle was determined with Muse® Cell Cycle Assay Kit (Luminex® Austin, Texas, United States). Cells (0.3 × 10^6^) were seeded on 6-well plates and cultured in standard conditions. The following day, cells were treated with experimental media for 24 h. After incubation time, cells were harvested and then cell cycle assay was conducted following manufacturer’s instructions (The results are shown as the percentage of cells in G0/G1, S and G2/M phase. The experiments was conducted in triplicate.

### DNA damage

To determine DNA damage in cells we used Muse® Multi-Color DNA Damage Kit (Luminex® Austin, Texas, United States). For this experiment, cells (0.25 × 10^6^) were seeded on 6-well plates and reached to get 90% confluence. Next, cells were treated as described above. After 24 h cells were harvested and DNA damage test was performed according to manufacturer’s instructions in three independent replicates.

### DAPI staining

Cells (15 × 10^3^) were seeded on 96-well plates and cultured in standard conditions. Then, cells were treated with DON and LY294002 and with their combination for 24 h. Next, the cells were washed two times in dPBS, fixed with 100% iced methanol (10 min), washed with dPBS, permabilized in 0.1% Triton X-100 in PBST, washed two times in dPBS (2 min) and stained with DAPI (Sigma Aldrich, St. Louis, MO, USA) for 1 min, again washed in dPBS two times and visualized at a FLoid Cell Imaging Station (Thermo Fisher Scientific Inc, Waltham, MA, USA).

### Confocal microscopy

Cells were seeded on 8-well chamber slide (Nunc™ Lab-Tek™ II Chamber Slide™ System/ Thermo Fisher Scientific Inc, Waltham, MA, USA) and reached to get 80% confluence. Then, cells were treated with DON and LY294002 for 24 h. After incubation time, the experimental medium was discarded and fixed with 70% iced methanol for 15 min in freezer. Next, cells were washed three times in dPBS, blocked (5% FBS, 0.3% Triton X-100 in dPBS 1X) for 1 h and then incubated in primary antibodies against LC3A/B (1:100, 1% BSA, 0.3% Triton X-100 in dPBS 1X. #12,741, Cell Signaling Technology) overnight. The next day, cells were washed in dPBS three times and incubated 1.5 h with secondary antibodies (1:400, Alexa Fluor Plus® 488, goat anti-rabbit #A32731, Thermo Fisher Scientific Inc, Waltham, MA, USA). Then, cells were washed three times in dPBS, walls were removed and slide was sticked down using Fluoroshield™ with DAPI (F6057, Sigma, St. Louis, MO, USA). Olympus iXplore SpinSR ScanR was used for visualization (Olympus, Tokyo, Japan).

### Real time quantitative polymerase chain reaction (RTqPCR)

Cells were seeded on 60 mm Petri dishes and allowed to reach 90% confluence. Then, cells were treated with the experimental medium as described above for 24 h. Cells were harvested using TRIzol Reagent (Thermo Fisher Scientific Inc, Waltham, MA, USA) and RNA was isolated following manufactuer’s instructions. The concentration of RNA was determined with a BioDrop spectrophotometer (BioDrop, Cambridge, UK). cDNA was synthesized from 5 µg of total RNA using ImProm RT-IITM reverse transcriptase (Promega, Madison, WI, USA) following manufacturer’s instructions. A LightCycler 96 (Roche, Basel, Switzerland) was used to conduct RT-qPCR reaction (2 µl of cDNA). Primer-BLAST (National Institutes of Health) was used to design primers (Supplementary file 1). The Human Reference RNA (Stratagene, San Diego, CA, USA) was used as a calibrator for each reaction. Ribosomal protein S17 (*RPS17*), ribosomal protein P0 (*RPLP0*), and histone H3.3A (*H3F3A*) were used as a reference genes. The melting curve analysis were performed for each reactions to confirm specificity of received product. The ΔΔCt method was used to analyze the obtained data. The experiment was carried out in duplicate with three independent replications.

### Western blot

Cells (2 × 10^6^) were seeded on 100-mm Petri dishes. The next day, cells were treated with DON and LY294002 as described above. After 24 h cells were harvested and frozen in − 80 °C. Protein was isolated using RIPA buffer with PMSF, protease and phosphatase inhibitor cocktails (Sigma-Aldrich, St. Louis, MO, USA). The concentration of protein was measured with DirectDetect® (Merck Millipore, Burlington, MA, USA). 30 µg of protein was used for electrophoresis (separation 12% polyacrylamide gel-120 V) and wet transfer (PVDF membranes, 100 V, 400 mA, 70 min). Then, membranes were blocked with 5% fat-free milk in TBST buffer for 1 h, washed three times in TBST for 5 min and then incubated overnight (4 °C) with primary antibodies. After incubation, the membranes were washed in TBST buffer as described above and the secondary (1:15,000) antibodies were added for 4 h (4 °C). Then, membranes were ones again washed in TBST, next, bands were visualized with Novex® AP Chromogenic Substrate (BCIP/NBT) (Thermo Fisher Scientific Inc, Waltham, MA, USA).

### Statistical analysis

Statistical analysis were performed using GraphPad Prism software. One-Way ANOVA was used to calculate statistical significance. The results were expressed as the mean ± SE. *P* value less than 0.05 was considered as statistically significant.

## Results

### DON decreases viability of PNT1A cells in a dose- and time-dependent manner

**Fig. 1:**
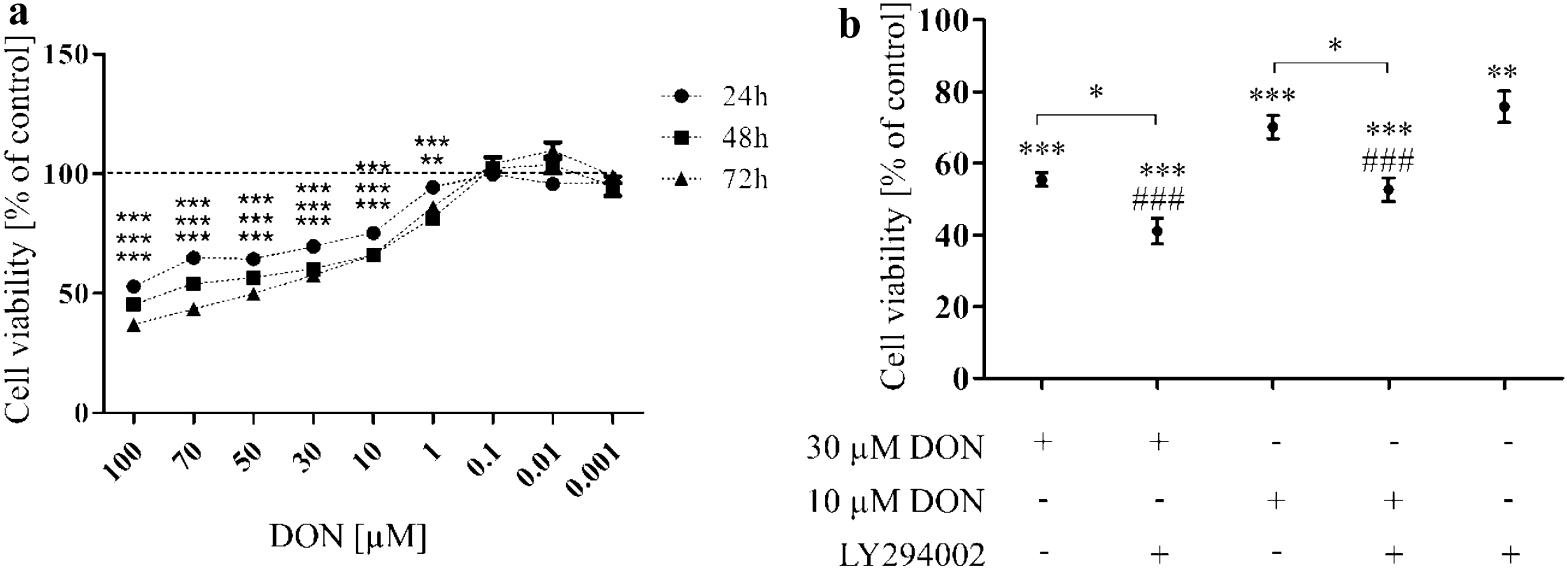
DON decreases viability of PNT1A cells in a dose- and time- dependent manner. **a** Dose- and time-dependent effect of DON on PNT1A cell viability. **b** The influence of PI3K inhibitor on DON- treated PNT1A cell viability. The viability of cells was evaluated with AlamarBlue assay. One-way ANOVA was used for statistical analysis of variances. *P* < 0.05 was considered as statistically significant. The results are presented and mean ± SE of % of control (non- treated cells).*p < 0.05, **p < 0.01, ***p < 0.001 as compared to control or indicated value, ###*p* < 0.001 as compared to Cnt + LY. DON-deoxynivalenol, Cnt- control, LY- LY294002, PI3K inhibitor

Firstly, the time- and dose-dependent effect of DON on cells viability was evaluated. We observed that DON in a dose- and time- dependent manner significantly decreased viability of prostate epithelial cells **(**Fig. 1a). Based on these results two doses of DON (30 and 10 µM) were chosen for the rest of experiments. Next, the viability of cells was once again evaluated with the chosen doses of DON and LY294002 to check how PI3K inhibitor affects viability of prostate epithelial cells (Fig. 1b). As suspected blocking of PI3K signaling pathway in PNT1A cells caused a decrease in the viability observed for cells treated only with LY294002 (**p < 0.01) as well as DON + LY294002 in both tested doses (*p < 0.05).

### DON induces oxidative stress and DNA damage in prostate epithelial cells

**Fig. 2:**
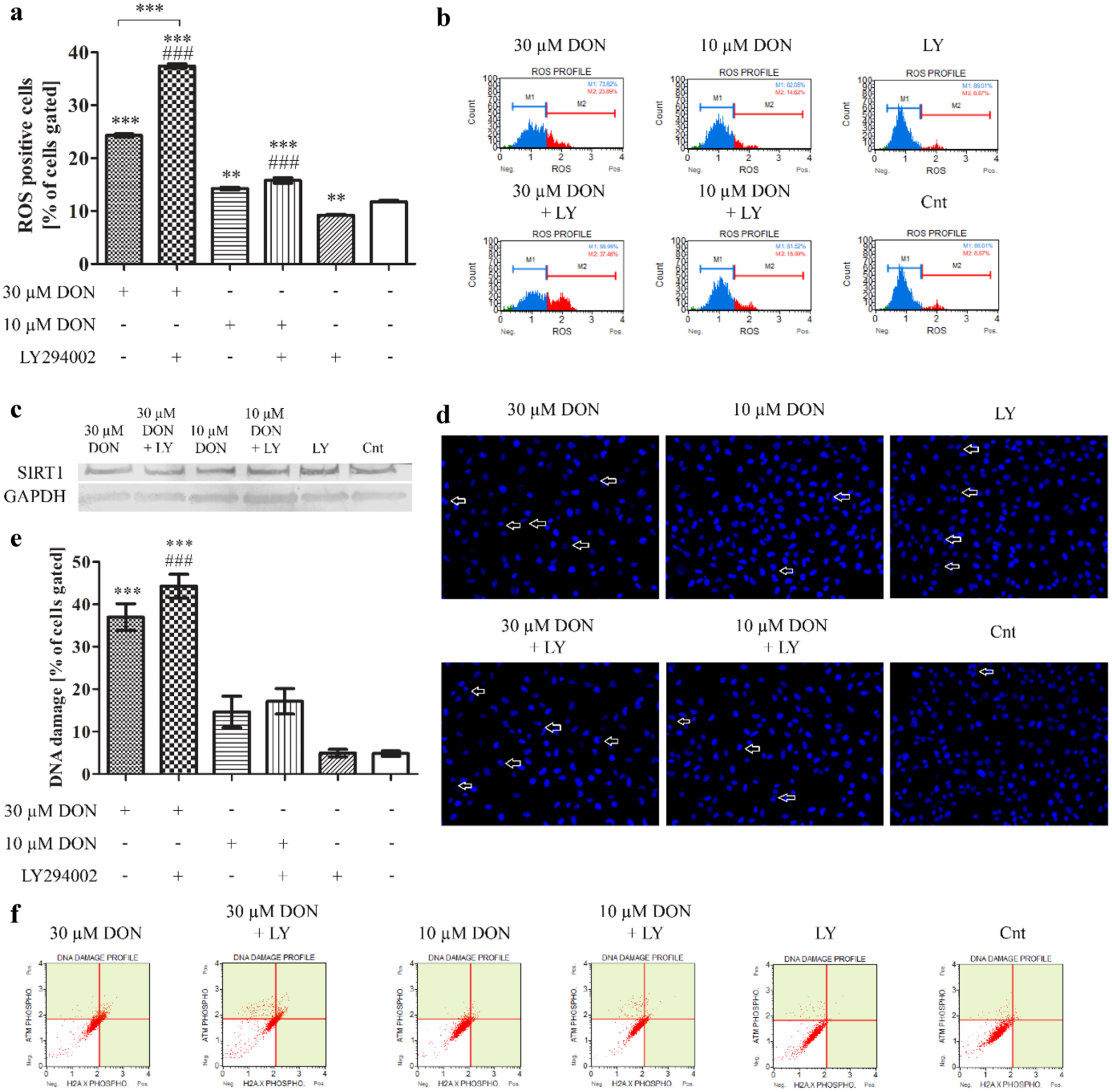
DON induces oxidative stress in PNT1A cells. **a** The analysis of ROS production in cells based on flow cytometry with representative results (**b**). **c** Western blot analysis of the expression of SIRT1. **d** DNA damage observed in DAPI staining. **e** DNA damage observed in flow cytometry (ATM and H2A.X activation) with representative plots (**f**). One-way ANOVA was used for statistical analysis of variances. *P* < 0.05 was considered as statistically significant. The results are presented and mean ± SE. *p < 0.05, **p < 0.01, ***p < 0.001 as compared to control or indicated value, ###*p* < 0.001 as compared to Cnt + LY. DON-deoxynivalenol, Cnt- control, LY- LY294002, PI3K inhibitor

DON is mainly associated with the induction of oxidative and ribotoxic stress (Savard et al. 2016). Thus, in the next step we evaluated if observed decreased viability of cells caused by DON is associated with induction of oxidative stress, DNA damage as well as regulation of cell cycle in cells (Fig. 2a, b). We observed that chosen doses of DON significantly increased the number of ROS positive cells (***p < 0.001, **p < 0.01 for 30 µM and 10 µM, respectively) as compared to control. Addition of LY294002 caused an increase in the number of ROS positive cells for both tested doses, however significant only for higher dose (***p < 0.001) as compared to DON treatment alone. In case of control cells, the number of ROS positive cells significantly decreased (**p < 0.01). The induction of oxidative stress was associated with changes in *HIF1α* expression which increased in a dose-dependent manner after DON treatment (***p < 0.001) as compared to not treated cells (Table 1). On the mRNA level, addition of LY294002 decreased the expression of *HIF1α* in DON-treated (**p < 0.001 and *p < 0.05) as well as control cells (not significant). Similar effect was observed in the expression of *NFR2* and its responsive gene *HMOX1*, as a first signaling pathway responding to the induction of oxidative stress in cells. A decrease in sirtuin 1 (SIRT1) expression was also observed (Fig. 2c).

**Table 1:** The expression of genes associated with oxidative stress obtained in RTqPCR

Gene	30 µM DON	30 µM DON + LY294002	10 µM DON	10 µM DON + LY294002	Cnt + LY294002	Cnt
*HIF1α*	2.423^***^	1.743^###,a^	2.078^***^	1.521^###,a^	0.598	0.984
*NRF2*	2.938^**^	1.256^a^	2.848^**^	1.301^a^	1.708	2.024
*HMOX1*	1.453^***^	1.133^###^	1.027	1.117^###^	0.400	0.740
*SIRT1*	7.814^***^	6.836^###^	5.285^***^	5.228^###^	1.422	2.126

The results are expressed as mean value of at least 4 replicates. One-way ANOVA was used for statistical analysis. *P* < 0.005 was considered as statistically significant. ^**^p < 0.01, ^***^p < 0.001 as compared to control, ^###^*p* < 0.001 as compared to Cnt + LY294002, ^a^*p* < 0.001 as compared to DON + LY294002. DON- deoxynivalenol, Cnt- control, *HIF1α*- hypoxia inducible factor 1 subunit alpha; *NRF2*- Nuclear factor erythroid 2-related factor 2, *HMOX*1- heme oxygenase 1, *SIRT1*- sirtuin 1

The increase in the induction of oxidative stress was associated with DNA damage in case of higher dose of DON (30 µM) and similar effect was observed after addition of PI3K inhibitor (Fig. 2 d, e, f). For lower dose of DON and control cells no such effect was observed indicating no DNA damage in cells. The DNA damage of cells was also visible in DAPI staining as fragmented nuclei of cells.

**Fig. 3:**
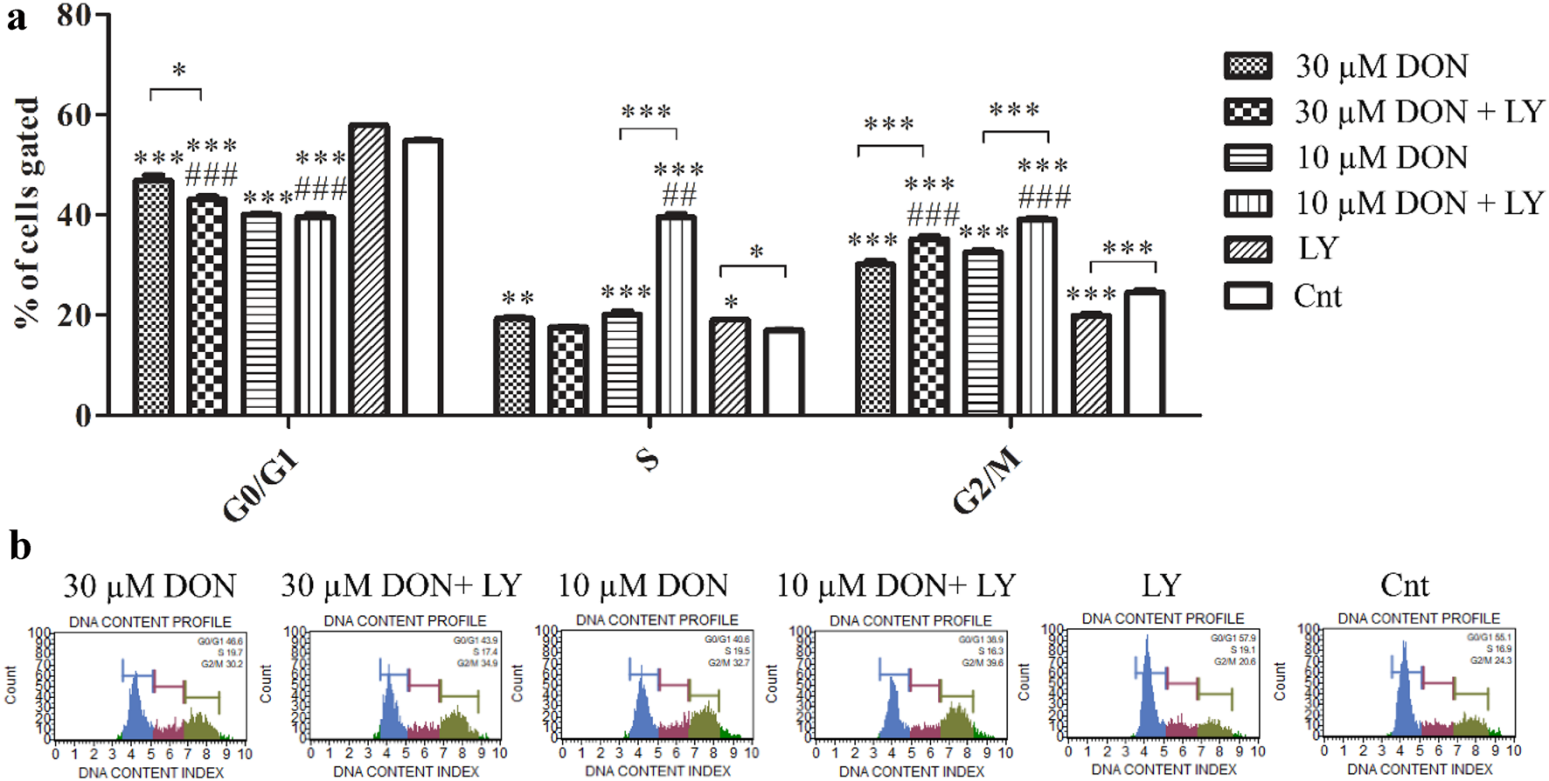
DON induces G2/M cell cycle arrest. **a** The analysis distribution of cells during cell cycle with representative plots (**b**). One-way ANOVA was used for statistical analysis of variances. *P* < 0.05 was considered as statistically significant. The results are presented and mean ± SE. *p < 0.05, **p < 0.01, ***p < 0.001 as compared to control or indicated value, ##*p* < 0.01, ###*p* < 0.001 as compared to Cnt + LY. DON-deoxynivalenol, Cnt- control, LY- LY294002, PI3K inhibitor

The observed induction of oxidative stress was associated with cell cycle arrest in G2/M cell cycle phase (Fig. 3a, b). The significant increase in the number of cells in G2/M cell cycle phase was observed for both tested doses (***p < 0.001). Addition of PI3K inhibitor to DON-treated cells significantly increased the number of cell in G2/M cell cycle phase (***p < 0.001). The increase was associated with significant decrease in the number of cell in G0/G1 cell cycle phase. Interestingly, such effect was not observed for control cells, indicating that decrease in G0/G1 is characteristic for DON treatment. Interestingly, for the lower dose of DON and LY294002 treatment, a significant increase in the number of cells in S cell cycle phase was observed, indicating a different mechanism of action of lower and higher tested dose of DON in prostate epithelial cells.

The cell cycle arrest in G2/M cell cycle phase was also visible in *CDC2* and *CCNB1* expression (Table 2). The *CDC2* expression was increased after DON treatment in both doses, whereas addition of LY294002 decreased significantly its expression as compared to DON treatment alone (**p < 0.01 and ***p < 0.001 for 30 µM and 10 µM, respectively). For control cells not significant increase was observed. The expression of *CCNB1* was also increased after DON treatment and decreased significantly in case of DON + LY294002 treatment as compared to DON treatment alone (***p < 0.001). Similar changes in the expression of the main regulator of cell cycle *CDKN1A* were observed.

**Table 2:** The expression of genes associated with oxidative stress obtained in RTqPCR

Gene	30 µM DON	30 µM DON + LY294002	10 µM DON	10 µM DON + LY294002	Cnt + LY294002	Cnt
*CDC2*	7.041^***^	3.963^###,aaa^	6.465^***^	4.042^###,aaa^	1.650	1.297
*CCNB1*	2.547^***^	0.9850^aaa^	2.448^***^	1.220^#,aaa^	0.6617^*^	1.222
*CDKN1A*	2.684^***^	1.890^a^	2.275^***^	1.717^a^	1.796^**^	1.107

The results are expressed as mean value of at least 4 replicates. One-way ANOVA was used for statistical analysis. *P* < 0.005 was considered as statistically significant. *p < 0.05, **p < 0.01, ***p < 0.001 as compared to control, # *p* < 0.05, ^###^*p* < 0.001 as compared to Cnt + LY294002, ^a^*p* < 0.05, ^aaa^*p* < 0.001 as compared to DON + LY294002. DON- deoxynivalenol, Cnt- control, *CDC2*- cyclin dependent kinase 1, *CCNB1*- cyclin B1, *CDKN1A*- cyclin dependent kinase inhibitor 1A

### DON induces apoptosis in PNT1A cells

**Fig. 4:**
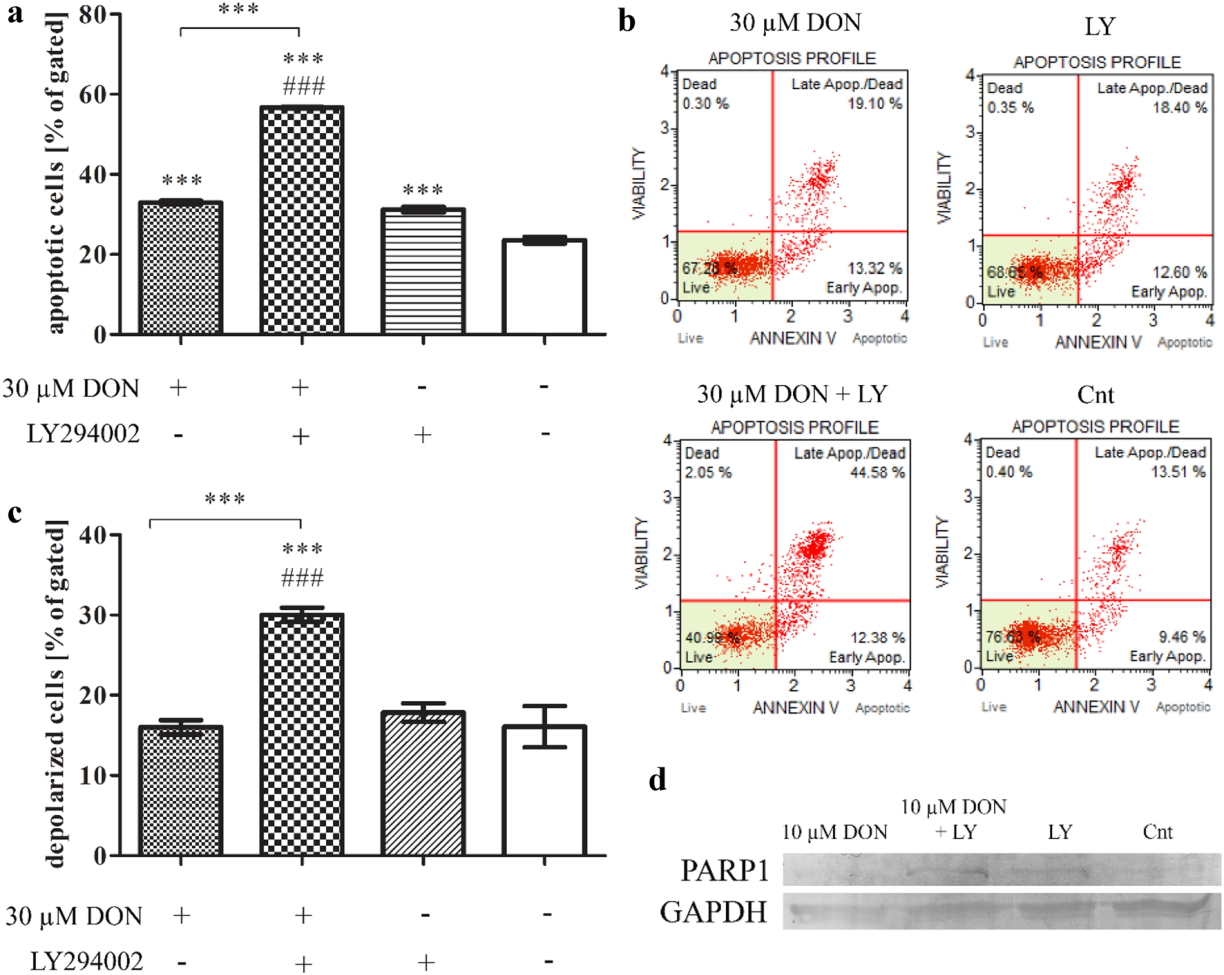
DON induces apoptosis in PNT1A cells. **a** The analysis of apoptotic cells based on flow cytometry with representative results (**b**). **c** The analysis of mitochondrial potential obtained in flow cytometry. **d** The expression of PARP1 in Western blot analysis. One-way ANOVA was used for statistical analysis of variances. *P* < 0.05 was considered as statistically significant. The results are presented and mean ± SE. ***p < 0.001 as compared to control or indicated value, ###*p* < 0.001 as compared to Cnt + LY. DON-deoxynivalenol, Cnt- control, LY- LY294002, PI3K inhibitor

It was suggested previously that DON might induce both authophagy and apoptosis in cells, dependently on it concentration (Gu et al. 2019). Based on our previous results presented above, we postulated that higher concentration of DON induces apoptosis in PNT1A cells. Staining with annexin V confirmed our assuption (Fig. 4a, b): a statistically significant increase in the number of apoptotic cells (***p < 0.001) as compared to control was observed. An addition of LY294002 significantly increased the number of apoptotic cells, indicating that observed decrease in cells viability was caused by apoptosis, however DON itself did not trigger increased depolarization of mitochondria in cells (Fig. 4C), whereas blocking of PI3K did it (***p < 0.001).

The induction of apoptosis by DON was associated with increased expression of *BAX* and *TP53*, which expression was significantly reduced after DON + LY294002 treatment (***p < 0.001) (Table 3). We also observed increased expression of *CASP3* and decreased expression of *PARP1*. On the protein level the expression of PARP1 (Fig. 4d) was significantly increased after DON + LY294002 treatment as compared to DON and control treatments.

**Table 3:** The expression of genes associated with apoptosis obtained in RTqPCR

Gene	30 µM DON	30 µM DON + LY294002	Cnt + LY294002	Cnt
*BAX*	1.496	0.7167^aaa^	0.8349	1.212
*TP53*	8.668	3.826^aaa^	2.936^***^	8.129
*CASP3*	4.022^***^	3.849^###^	1.215	1.478
*PARP1*	0.8093^**^	0.6691^##^	0.9744	1.173

The results are expressed as mean value of at least 4 replicates. One-way ANOVA was used for statistical analysis. *P* < 0.005 was considered as statistically significant. **p < 0.01, ***p < 0.001 as compared to control, ##*p* < 0.01, ^###^*p* < 0.001 as compared to Cnt + LY294002, ^aaa^*p* < 0.001 as compared to DON + LY294002. DON- deoxynivalenol, Cnt- control, *BAX*- Bcl-2 associated X protein, *TP53*- tumor protein p53, *CASP3-* caspase 3, *PARP1*- Poly (ADP-ribose) polymerase 1

### DON induces autophagy in prostate epithelial cells

**Fig. 5:**
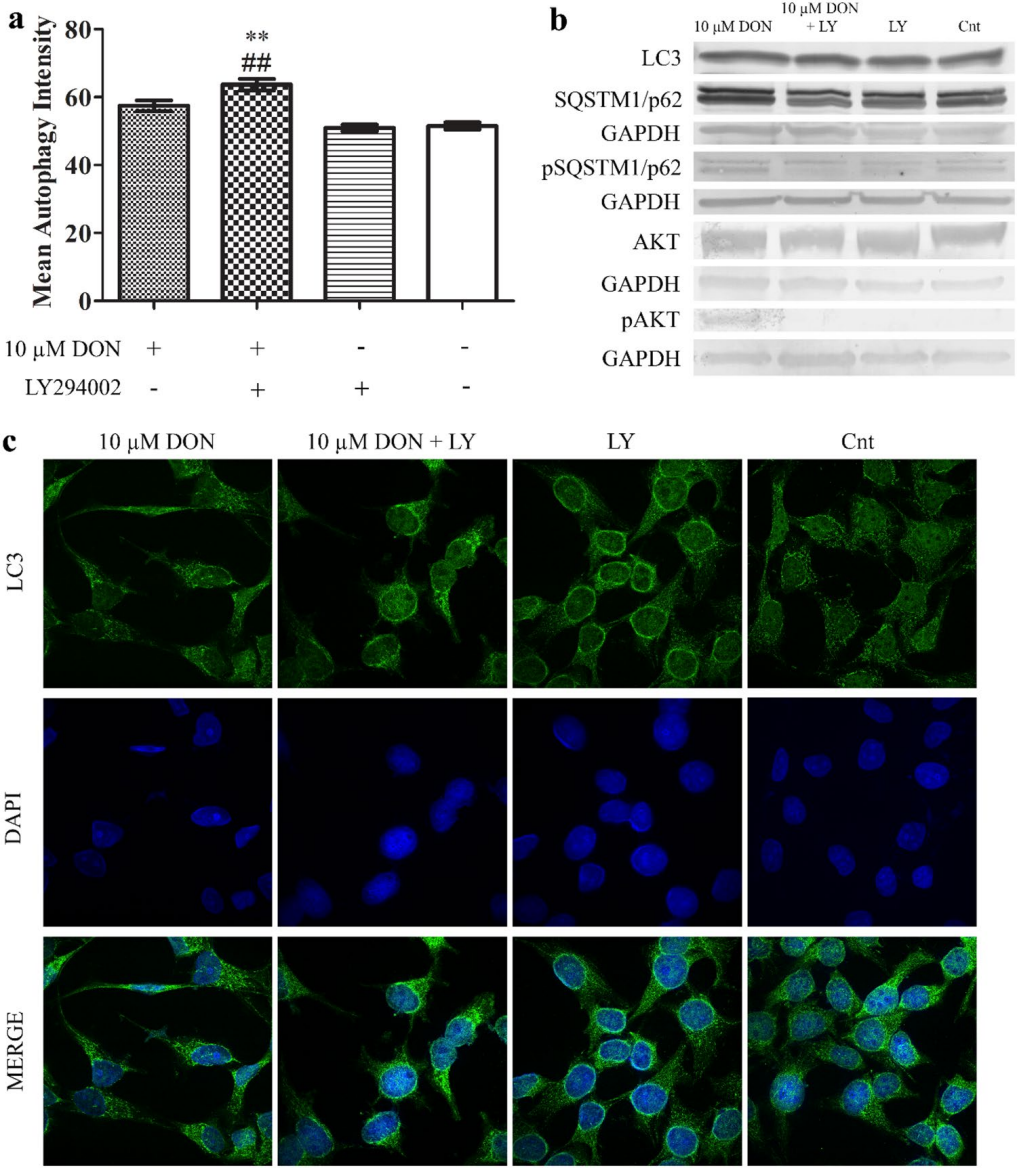
DON induces autophagy in PNT1A cells. **a** The analysis of the induction of autophagy based on flow cytometry. **b** The representative results of Western blot analysis. **c** Localization and expression of LC3 obtained during confocal microscopy. One-way ANOVA was used for statistical analysis of variances. *P* < 0.05 was considered as statistically significant. The results are presented and mean ± SE. **p < 0.01 as compared to control or indicated value, ##*p* < 0.01 as compared to Cnt + LY. DON-deoxynivalenol, Cnt- control, LY- LY294002, PI3K inhibitor, LC3- Microtubule-associated protein 1A/1B-light chain 3, SQSTM1/p62- sequestosome-1, GAPDH- glyceraldehyde 3-phosphate dehydrogenase, p SQSTM1/p62- phosphorylated sequestosome-1, AKT- protein kinase B, pAKT- phosphorylated protein kinase B

It was suggested previously that DON might induce apoptosis via induction of autophagy (Gu et al. 2019). We observed that the lower tested concentration of DON also induced autophagy in PNT1A cells (Fig. 5a). The induction of autophagy was visible in the changes in the expression and localization of LC3 protein observed during confocal microscopy (Fig. 5c). We also observed decreased expression of p62 and increased expression of phospho-p62 similarly to the expression of LC3I and LC3 II on the protein level (Fig. 5b). In all experiments addition of LY294002 increased the induced by DON autophagy in cells. The activation of Akt as its phosphorylation was observed in both tested doses of DON (Fig. 3), diminished by LY294002. An increased expression of *BECN1*, *MAP1LC3B* and *ATG7* was directly associated with the observed induction of autophagy in cells (Table 4).

**Table 4:** The expression of genes associated with apoptosis obtained in RTqPCR

Gene	10 µM DON	10 µM DON + LY294002	Cnt + LY294002	Cnt
*BECN1*	0.3750^*^	0.2300^aa^	0.1900	0.2525
*MAP1LC3B*	5.712^***^	5.148^##^	2.735	2.437
*ATG7*	0.9500	0.4733^aa^	0.4367	0.7383

The results are expressed as mean value of at least 4 replicates. One-way ANOVA was used for statistical analysis. *P* < 0.005 was considered as statistically significant. *p < 0.05, ***p < 0.001 as compared to control, ##*p* < 0.01as compared to Cnt + LY294002, ^aa^*p* < 0.01 as compared to DON + LY294002. DON- deoxynivalenol, Cnt- control, *BECN1*- beclin 1, *MAP1LC3B*- microtubule-associated protein 1A/1B-light chain 3, *ATG7-* autophagy related 7

## Discussion

Prostate cancer is one of the most common cancers and its incidence is increasing with age. It is believed that lifestyle and diet might play a role, based on the assumption that Asian men with the lowest incidence of prostate cancer, after moving to Western countries or USA present a similar incidence of prostate cancer to other men living there (Shimizu et al. 1991). Present in the diet mycotoxins and their effect on human and animal health is under constant monitoring, based on the research reporting their harmful effect on animal and human (Knutsen et al. 2017). DON is one of the most common mycotoxin with reported toxic effect on human and animal. It ingested in high doses causes vomiting and diarrhea, whereas in low concentrations weight loss (Rotter et al. 1996). Although DON is not reported to directly being a cause of gene mutations in cells and consequently not considered as carcinogen in humans due to not sufficient data, it affects protein synthesis, cells proliferation and death as well as cells motility and invasion (Gu et al. 2019).

More than 100 substrates of Akt are participating in the basic cellular processes in cells: proliferation, protein synthesis, metabolism and migration and as a consequence modulation of PI3K/Akt signaling pathway is commonly observed in many tumors, including prostate. Previous studies suggested that observed cytotoxicity induced by DON might be associated with modulation of PI3K/Akt (Zhao et al. 2020). The effect of DON on prostate cancer cells was evaluated by us previously, however the effect on normal prostate epithelial cells is not known. Thus, this study evaluated the effect of DON on normal prostate epithelial cells with a focus on the involvement of PI3K/Akt signaling pathway. The results of this study showed that DON induces reduction of cell viability, oxidative stress and DNA damage in cells. The induction of oxidative stress was associated with cell cycle arrest in G2/M cell cycle phase. The induction of oxidative stress in cells was previously observed by Zhang et al. in porcine kidney cell line (Zhang et al. 2018) as well as prostate cancer cells (Habrowska-Górczyńska et al. 2019). The observed induction of oxidative stress was present for relatively high doses of DON, indicating that normal prostate epithelial cells are not so sensitive to DON-induced cytotoxicity.

The observed cell cycle arrest in G2/M cell cycle phase is also characteristic for DON-induced oxidative stress in cells, observed previously in HepG2 cells (Yuan et al. 2018), human gastric epithelial GES-1 (Yang et al. 2018), porcine epithelial cell line J2 (Liao et al. 2017), chicken embryo fibroblast DF-1 (Li et al. 2014) and other studies. The arrest in G2/M cell cycle phase was associated with the modulation of the expression of *CCNB1*, *CDC2* and *CDKN1A* genes responsible for regulation of cell cycle. The changes in the expression of cell cycle-related cytokines was observed by Dai et al. in mouse endometrial stromal cells where DON caused a decrease in the expression of complex cyclin B1 and cdc2 (Dai et al. 2017). In our study, a contradictory effect was observed: DON itself significantly increased expression of the G2/M cell cycle complex and p21, whereas addition of PI3K blocker caused a reduction in the expression indicating that G2/M cell cycle arrest caused by DON in PNT1A cells is associated with PI3K/Akt signaling pathway.

Autophagy might play a dual role in cells, on the one hand its protects against harmful conditions to improve cell survival, but on the other hand it might induce programmed cell death in cells (Jing and Lim 2012). DON was previously reported to induce autophagy in porcine epithelial cells (Gu et al. 2019), bladder cancer cells (Del Favero et al. 2021), murine skin (Mishra et al. 2020), rat adrenal gland cells (Wang et al. 2020). In most of the studies the induction of autophagy was associated with toxicity of DON and modulated by PI3K/Akt signaling pathway. Moreover, the association of autophagy and apoptosis caused by DON was suggested previously (Wang et al. 2020). In this study the induction of autophagy in cell was associated with increased expression of beclin-1, p62, LC3 and *ATG7.* The observed significant decrease in the expression of autophagy markers after adding LY294002, confirmed our assumption that DON induces autophagy in cells via PI3K/Akt signaling pathway. This observation is in line with other research studies presenting DON-induced autophagy in cells (Gu et al. 2019; Wang et al. 2020). As reported by Peng et al. DON-induced apoptosis is associated with increased expression of caspase 3 (Peng et al. 2020) and increased expression of Bax (Hou et al. 2021). The observed in PNT1A cells apoptosis was associated with modulation of the expression of Bax and caspase 3 similarly to previous studies. We observed that DON-induced autophagy was also associated with modulation of mitochondria membrane potential, similarly to DON-induced apoptosis in prostate cancer cells (Habrowska-Górczyńska et al. 2019). Blocking of PI3K/Akt signaling pathway resulted in the increased number of apoptotic cells indicating that PI3K/Akt protects cells against DON-induced apoptosis. Similar modulation of PI3K/Akt pathway in prostate cells was observed by Sun et al. with arctigenin (Sun et al. 2021).

PI3K/Akt acts as important upstream modulator of Nrf2/HO-1 (Wang et al. 2008). Regulation of Nrf2 signaling pathway in the response to DON-induced toxicity was suggested before (Zhou et al. 2021). Nrf2/Keap1 serves as the major antioxidant response in cells and was reported to protect cells against drugs and mycotoxins (Ndlovu et al. 2021). Similarly, we observed that in case of normal prostate epithelial cells the expression of *NRF2* was significantly increased after DON treatment as compared to control, whereas simultaneous treatment with LY294002 decreased it. Similar effect was observed in HepG2 cells where the decrease in the expression of Nrf2 was associated with increase in its phosphorylated form (Ndlovu et al. 2021). The increased expression of Nrf2 associated with oxidative stress induced by DON was also observed in bovine mammary epithelial cells by Zhang et al. (Zhang et al. 2021). The activation of Nrf2/HO-1 was postulated as a main response element in other cells exposed to DON e.g. DON-induced neurotoxicity (Zhang et al. 2020) or generally one of the basic cell signaling element in the response to mycotoxins (Kozieł et al. 2021).

Taken together, this study for the first time reports that DON in high doses might induce oxidative stress and in a consequence autophagy and apoptosis in normal prostate epithelial cells via modulation of PI3K/Akt signaling pathway.

## Supplementary Information

Below is the link to the electronic supplementary material.

Supplementary file1 (DOCX 14 kb)

## Data Availability

Data available upon request.
