# Supplementary material for: Deoxynivalenol induces apoptosis and autophagy in human prostate epithelial cells via PI3K/Akt signaling pathway
Source: Arch Toxicol. 2021 Oct 22;96(1):231–41. doi: 10.1007/s00204-021-03176-z (PMC8748346; doi:10.1007/s00204-021-03176-z)
Supplement: Supplementary file 1 — Supplementary file1 (DOCX 14 kb) [file 204_2021_3176_MOESM1_ESM.docx]

**Supplementary file 1**

**Table 1. The primers used in RTqPCR.** *ATG7-* autophagy related 7, *BAX*- Bcl-2 associated X protein, *BECN1*- beclin 1, *CASP3-* caspase 3, *CDC2*- cyclin dependent kinase 1, *CDKN1A*- cyclin dependent kinase inhibitor 1A, *CCNB1*- cyclin B1, *HIF1α*- hypoxia inducible factor 1 subunit alpha, *HMOX*1- heme oxygenase 1, *MAP1LC3B*- microtubule-associated protein 1A/1B-light chain 3, *NRF2*- Nuclear factor erythroid 2-related factor 2, *PARP1*- Poly (ADP-ribose) polymerase 1, *SIRT1*- sirtuin 1, *BAX*- Bcl-2 associated X protein, *TP53*- tumor protein p53, *H3F3A-* histone H3.3, *RPLP0*- ribosomal Protein Lateral Stalk Subunit P0, *RPS17*- ribosomal protein s17.

| **Gene** | **Sequence (5’-3’)** | **Product size [bp]** |
| --- | --- | --- |
| *ATG7* | For TTGGCATGAGTTGACCCAGAAGAAG  Rev AGCCCAGCAGAGTCACCATTGTA | 99 |
| *BAX* | For AGAGGTCTTTTTCCGAGTGGCAGC  Rev TTCTGATCAGTTCCGGCACCTTG | 137 |
| *BECN1* | For CCATGCAGGTGAGCTTCGTGTG  Rev GTTTCGCCTGGGCTGTGGTAAG | 129 |
| *CASP3* | For GGAATATCCCTGGACAACAGTT  Rev TTGCTGCATCGACATCTGT | 130 |
| *CDC2* | For TTTTCAGAGCTTTGGGCACT  Rev AGGCTTCCTGGTTTCCATTT | 100 |
| *CDKN1A* | For GACAGATTTCTACCACTCCAA  Rev CTGAGACTAAGGCAGAAGAGT | 134 |
| *CCNB1* | For ACCTATGCTGGTGCCAGTG  Rev GGCTTGGAGAGGCAGTA | 128 |
| *HIF-1 α* | For TTACTCATCCATGTGACCATGA  Rev AGTTCTTCCTCGGCTAGTTAG | 140 |
| *HMOX1* | For CAGCTCCTGCAACTCCTCAAA  Rev TTCTTCACCTTCCCCAACATTG | 165 |
| *MAP1LC3B* | For TCACAGAACCCGCCGCCTTT  Rev TACCCTGCGTTTGTGCCAACTG | 140 |
| *NRF2* | For GTCACATCGAGAGCCCAGTC  Rev ACCATGGTAGTCTCAACCAGC | 193 |
| *PARP1* | For TCTTCAAGAGCGATGCCTATT  Rev TGAGGTAAGAGATTTCTCGGAA | 129 |
| *SIRT1* | For CATAGACACGCTGGAACA  Rev GCTTCACAGTCAACTTTGTA | 108 |
| *TP53* | For TTGGAACTCAAGGATGCC  Rev TTTATGGCGGGAGGTAGA | 102 |
| *H3F3A* | For AGGACTTTAAAAGATCTGCGCTTCCAGAG  Rev ACCAGATAGGCCTCACTTGCCTCCTGC | 74 |
| *RPLP0* | For ACGGATTACACCTTCCCACTTGCTAAAAGGTC  Rev AGCCACAAAGGCAGATGGATCAGCCAAG | 69 |
| *RPS17* | For AAGCGCGTGTGCGAGGAGATCG  Rev TCGCTTCATCAGATGCGTGACATAACCTG | 87 |
